# A New, Biomimetic Collagen–Apatite Wound-Healing Composite with a Potential Regenerative and Anti-Hemorrhagic Effect in Dental Surgery

**DOI:** 10.3390/ma15248888

**Published:** 2022-12-13

**Authors:** Barbara Kolodziejska, Lukasz Pajchel, Anna Zgadzaj, Joanna Kolmas

**Affiliations:** 1 Department of Analytical Chemistry and Biomaterials, Faculty of Pharmacy, Medical University of Warsaw, ul. Banacha 1, 02-097 Warsaw, Poland; 2 Department of Environmental Health Science, Faculty of Pharmacy, Medical University of Warsaw, Banacha 1, 02-097 Warsaw, Poland

**Keywords:** composite biomaterials, carbonate hydroxyapatite, zinc, magnesium, tranexamic acid, drug delivery, wound healing

## Abstract

The aim of this work was to obtain and characterize composite biomaterials containing two components, namely carbonated hydroxyapatite, which was substituted with Mg^2+^ and Zn^2+^ ions, and natural polymer–collagen protein. The following two different types of collagen were used: lyophilized powder of telocollagen from bovine Achilles tendon and atelocollagen solution from bovine dermis. The obtained 3D materials were used as potential matrices for the targeted delivery of tranexamic acid for potential use in wound healing after tooth extractions. Tranexamic acid (TXA) was introduced into composites by two different methods. The physicochemical analyses of the obtained composites included Fourier-transform infrared spectroscopy (FT-IR), inductively coupled plasma–optical emission spectroscopy (ICP-OES), transmission electron microscopy (TEM), scanning electron microscopy (SEM), powder X-ray diffraction (PXRD), release kinetics tests, swelling test, and cytotoxicity assays. The studies showed that the proposed synthetic methods yielded biomaterials with favorable physicochemical properties, as well as the expected release profile of the drug and ions from the matrices.

## 1. Introduction

The extracellular matrix of bone is a composite material consisting primarily of calcium phosphate apatite, collagen type I, and water. The chemical composition of osseous tissue depends on hormonal, nutritional, and mechanical factors, as well as on the bone’s location. It is assumed that 60–70 wt.% of bone tissue is in mineral phase, 20–30 wt.% is an organic matrix, and the rest is water [1,2]. Mineral fraction is formed by thin, plate-like nanocrystals of calcium- and hydroxyl-ion-deficient hydroxyapatite, enriched with a variety of different ions (e.g., Mg^2+^, K^+^, Na^+^, Mn^2+^, CO_3_^2−^, SiO_4_^4−^) [3,4]. The apatite nanocrystals are oriented with their *c-axis* parallel to each other and aligned with collagen molecules [5].

Bone tissue is known for its remodeling ability. However, in special cases of bone defects, such as trauma, osteomyelitis, osteosarcoma, or complicated tooth extraction, when natural bone regeneration fails, some clinical interventions are required [1].

Currently, biomaterial engineering is still looking for the ideal bone-substitute material, which should exhibit properties such as biocompatibility, durability, appropriate porous structure, osteoconduction, bioactivity, etc. [6]. Therefore, to achieve structural integrity and great tensile strength, selecting an ideal biomaterial is a challenge [7]. Collagen–apatite composites have attracted extensive attention from researchers due to their great similarity to bone tissue and very promising properties [4,8,9,10,11]. So far, the composites have been obtained primarily using stoichiometric hydroxyapatite with the general formula Ca_10_(PO_4_)_6_(OH)_2_. However, HA in its stoichiometric form is characterized by poor bioactivity and weak bioresorbability; therefore, it does not fully simulate the properties of osseous apatite [12]. The high ease of ionic substitution in HA crystals enables the introduction of ions present in natural apatite, but it also yields the modification of ions with additional favorable properties.

In our work, HA was substituted with two main “impurities” of bone apatite, magnesium, and carbonates, as well as with zinc ions. Magnesium is required to maintain homeostasis in mineralized tissues. It stimulates osteoblasts and influences the secretion of parathyroid hormone (PTH) and vitamin D. Magnesium deficiency increases the activity of osteoclasts, which contributes to bone loss [13,14]. Approximately 5–8 wt.% carbonates are present in bone apatite. Carbonates significantly affect the morphology and physicochemical properties of apatite crystals: they increase the c-axial length, decrease the a-axial length, and improve bioresorbability and porosity [15,16].

In turn, zinc stimulates the process of osteoblastogenesis and the production of bone growth factors. It takes part in the mineralization of bone tissue and inhibits bone resorption [17,18,19].

It is worth noting that, during orthopedic surgery or several dental procedures, hemorrhages are a serious problem in surgery. Often in such situations, blood transfusions are performed, but it is known that they are associated with many complications, such as blood-borne infections, immune cross-reactions, or thromboembolic events [20,21]. A promising solution to this problem may be to offer a new function to the scaffold material—it can serve as a carrier for a hemostatic drug to shorten the bleeding and reduce blood loss. One of these drugs is tranexamic acid (TXA). Its mechanism of action is reversibly blocking lysine-binding sites of plasminogen, resulting in the conversion of plasminogen to plasmin being blocked, thereby preventing the dissolution of the fibrin clot. In this way, the antifibrinolytic effect is achieved [22,23]. The application of the drug directly at the site of action can significantly reduce both the therapeutic dose and the occurrence of unfavorable side effects [24]. It should not be forgotten that plasminogen is a very important factor in the wound-healing process. It leads to cytokine induction and intracellular-signaling events, resulting in an enhanced and early inflammatory response. TXA would be helpful in controlling the bleeding, but the inhibition of plasminogen results in poor angiogenesis in the healing area [25,26,27]. In our opinion, its use in minor procedures (e.g., dental) is justified because we want to quickly stop bleeding in a relatively small surgical area. Moreover, our goal is to quickly release TXA from the composite matrix. After the release of TXA, the material is supposed to act as a bone-substitute material, slowly releasing additional zinc and magnesium ions that have a positive effect on bone growth.

So far, there have been several studies on the local delivery of TXA by calcium-phosphate-based materials and there is scope for further development [28].

It should be mentioned that collagen (in the form of sponges) is also used as a topical hemostatic agent. It serves as a matrix of clot formation. Moreover, it plays the role of initiator of the coagulation cascade. The use of tranexamic acid in combination with collagen may be considered in the prevention of excessive blood loss [29,30,31].

The aim of this work is to develop a new, three-dimensional collagen/modified nanoapatite composite containing TXA. Due to the hemostatic effect of TXA and collagen, it could be potentially used as a wound dressing in dental surgery, i.e., socket grafting and alveolar bone preservation. The use of biomimetic apatite with zinc ions may induce a natural process of bone tissue regeneration, which is an important issue after tooth extraction [32]. 

## 2. Materials and Methods

### 2.1. Samples Preparation

The mineral fraction of the composite, consisting of carbonate hydroxyapatite containing magnesium and zinc ions, was synthesized using the wet precipitation method in an air atmosphere. Calcium nitrate (V) tetrahydrate (Ca(NO_3_)_2_·4H_2_O), diammonium carbonate ((NH_4_)_2_CO_3_), purchased from Chempur (Piekary Śląskie, Poland), and ammonium phosphate dibasic ((NH_4_)_2_HPO_4_), zinc nitrate hexahydrate (Zn(NO_3_)_2_·6H_2_O), magnesium chloride anhydrous (MgCl_2_), purchased from Sigma Aldrich (Bangalore, India), were used as the substrates for the synthesis. All the reagents were weighed out to obtain the compound with the nominal composition Ca_8,25_Zn_0,5_Mg_0,25_(PO_4_)_5_CO_3_OH and then dissolved separately in distilled water. The sources of phosphates and carbonates were slowly added to the sources of calcium, zinc, and magnesium, stirring constantly. The pH was adjusted to 11 using a concentrated ammonia solution, and the obtained suspensions were then mixed for two hours. The slurry was left to age for 7 days without being stirred. This process of aging is of great importance in ensuring biocompatibility and the mapping of the physicochemical properties of biological apatite. After that, the precipitate was washed several times with distilled water, filtered, and dried at a temperature of 100 °C for 24 h. Then, the material was crushed in an agate mortar to subject it to further tests and use in a composite synthesis. The powder was named mHA (mimetic hydroxyapatite). 

mHA and two different types of collagen were used as starting materials to obtain the composites. As collagen proteins, collagen type I from bovine Achilles tendon (Sigma Aldrich, St. Louis, MO, USA) and atelocollagen from bovine dermis (3 mg/mL) (Cosmo Bio Co., Tokyo, Japan), were used. Type I collagen was suspended in 1% acetic acid to obtain a 0.5% solution of collagen. Then, 10 mL of collagen slurry was mixed at 4 °C with 240 mg of the mHA powder using a mechanical stirrer until a satisfactory level of homogeneity was achieved. After that, the pH of the slurry was adjusted to 8 using a concentrated ammonia solution. The precipitate was washed several times with distilled water, frozen, and freeze-dried. Composites with atelocollagen were obtained identically, except for the preparation of the collagen suspension.

TXA (purchased from TCI Chemicals, Belgium) was added to both composites at two different stages of synthesis. The first way was to add 10 mg of TXA directly to the obtained suspension (before freezing). The other method consisted of soaking the obtained composites for one hour in a drug solution (0.3 mol/L) after lyophilization. Then, the composites were again frozen and lyophilized. All types of the obtained composites are presented in Table 1.

### 2.2. Analytical Methods

To confirm the identity of the obtained powder and composites, Fourier-transform infrared spectroscopy (FT-IR) studies were performed using a PerkinElmer Spectrum 1000 (Waltham, MA, USA) spectrometer. Transmission spectra were acquired in the 4000–400 cm^−1^ range at a spectral resolution of 2 cm^−1^ from KBr pellets using 30 scans. All of the obtained spectra were processed using GRAMS/AI 8.0 (Thermo Fisher Scientific, Waltham, MA, USA) and KaleidaGraph 3.5 (Synergy Software, Reading, PA, USA) software.

The phase composition of the synthesized powder was analyzed by powder X-ray diffraction (PXRD). The patterns were collected using a Bruker DX8 Discover diffractometer (Billerica, MA, USA) using (CuKα radiation *λ* = 1.54 Å), in the 2 theta range from 20° to 70°. The crystallite size was determined using the Scherrer formula:

$$d=\frac{0.94\lambda }{\beta \cos\theta },$$

where 

*d* is the crystallite size (nm),

*λ* is the X-ray wavelength (nm),

*β* is the line broadening at half the maximum intensity (radians), and

*θ* is the Bragg angle for the corresponding reflection (°).

To estimate the crystallite size along the *a* and *c* axes, reflections at approximately 26.1° and 39.5°, respectively, were chosen.

The microstructural features of the powder sample were studied using the high-performance JEM 1400 transmission electron microscope (TEM-JEOL Co., Tokyo, Japan, 2008), equipped with an 11-megapixel MORADA G2 TEM camera (EMSIS GmbH, Germany) under an accelerating voltage of 80 kV. The analyzed material was prepared by suspending the powder sample in 96% ethanol, then dropping it onto a copper grid, followed by air-drying.

The concentration of calcium, phosphorus, magnesium, and zinc was measured via inductively coupled plasma–optical emission spectroscopy (ICP-OES), using an Optima 3100 XL PerkinElmer spectrometer (Llantrisant, UK). The powder sample was dissolved in concentrated HNO_3_ (Suprapur, Sigma Aldrich, St. Louis, MO, USA) and diluted properly with deionized water. To calculate the carbonate content (types A + B), a method described previously by Clasen and Ruyter was used [33,34]. 

To determine the morphology of the composites, scanning electron microscopy (SEM-JSM-6390LV JEOL microscope, JEOL LTD., Tokyo, Japan) at a 20 or 30 kV accelerating voltage was chosen.

The in vitro cytotoxicity assessment was conducted. The neutral red uptake test was performed based on ISO 10993 guideline Annex A [35] with a BALB/c 3T3 clone A31 mammalian cell line (mouse embryonic fibroblasts from the American Type Culture Collection). The quantitative estimation of viable cells in the tested cultures was based on their neutral red uptake in comparison to the results obtained for the untreated cells. Dead cells had no ability to accumulate the dye in their lysosomes. The BALB/c 3T3 cells were seeded in 96-well microplates (15,000 cells/100 µL) in a DMEM (Lonza, Walkersville, MD, USA) culture medium (supplemented with 10% calf bovine serum, 100 IU/mL penicillin, and 0.1 mg/mL streptomycin) and incubated for 24 h (5% CO_2_, 37 °C, >90% humidity). At the end of the incubation, each well was examined under a microscope to ensure that the cells formed a confluent monolayer. Afterwards, the culture medium was replaced with the tested extracts. Extracts were prepared by incubating the tested materials in the cell culture medium (50 mg/mL) with a reduced serum concentration (5%) at 37 °C for 24 h with shaking and sterilization by filtration. Cells were treated with four dilutions of each extract in a two-fold dilution series for 24 h (three data points for each one). Subsequently, the treatment medium was removed. Cells were washed with PBS and treated with the neutral red medium for 2 h. Then, the medium was discarded, and cells were washed with PBS and treated with desorbing fixatives (ethanol and acetic acid in water solution). The amount of neutral red accumulated by cells was evaluated colorimetrically at 540 nm. Polyethylene film and latex were used as the reference materials (with no cytotoxicity and high cytotoxicity, respectively). The percentage of viable cells in each well was calculated by comparing its OD540 result with the mean result obtained for the untreated cells (incubated in the same conditions as the fresh culture medium). Samples were considered cytotoxic if they reduced cell survival to below 70% compared to the untreated cells (baseline cell viability). When BALB/c 3T3 cell viability did not decrease to under 70% in the whole range of tested dilutions of the samples, it was considered non-cytotoxic in this range of concentrations.

The swelling ratio of the obtained composites (OmHA1, OmHA2, AmHA1, and AmHA2) and the pure collagen sponge (1 cm × 1 cm × 1 cm) was measured by incubation in ultrapure water at 37 °C. Subsequently, the samples were withdrawn from the solution after soaking for 15, 30, 60, 180, and 360 min, and the surface adsorbed water was removed by filter paper. The swelled samples were then weighed immediately. The swelling ratio was definite as the ratio of weight increased (W-W0) to the initial weight (W0). Each sample was tested in triplicate.

The in vitro release of TXA from the composites was evaluated in Falcon 50 mL tubes. The studies were performed in a phosphate buffer (pH = 7.4). Each tube contained 250 mg of a specific type of composite immersed in 50 mL of the release medium in the bath shaker and stirred at 100 rpm at 37 °C before being incubated for seven days. Sample aliquots of 5 mL were withdrawn at regular time intervals (15 min, 30 min, 1 h, 3 h, 6 h). All the samples were filtered through a membrane syringe filter with a pore size of 0.8 μm. Each time, the volume of the medium taken for analysis was replaced with a new phosphate buffer portion. The samples were then analyzed by HPLC using chromatographic equipment, consisting of a Varian Prostar 210 isocratic pump (Palo Alto, CA, USA) and a Rheodyne 7725i injector (Cotati, CA, USA) with a 20 μL sample loop. Detection was performed by a Varian Prostar 325 UV detector using a detection wavelength of 220 nm. The chromatographic conditions and measurement procedures were previously described [36]. The LC column used was a 4.6 mm i.d. × 250 mm length XTerra RP 18 analytical column that was purchased from Waters (Milford, Ireland). The mobile phase consisted of anhydrous sodium dihydrogen phosphate, sodium lauryl sulfate, and triethyl amine (pH 2.5) mixed with methanol in a ratio (60:40, v/v). It was degassed by sonication before use. The flow rate of the mobile phase was maintained at 1 mL/min. HPLC analysis was conducted at 30 °C. Peak areas were measured for the quantitation of the TXA. Stock solutions of TXA 500 ppm were prepared by dissolving the appropriate amount in water. Calibration standards were prepared over a concentration range of 20, 50, 100, 250, and 500 ppm for TXA by the appropriate dilutions of the above-mentioned standard solution. Calibration standards were analyzed in triplicate for the calibration curve.

The in vitro release of zinc and magnesium ions was studied in the same manner as the TXA release test. However, a decision was made to extend the duration of the experiment, and the samples were withdrawn at the following intervals: 15 min; 30 min; 1 h; 3 h; 6 h; 12 h; 1 day; 2 days; and 7 days. The concentration of magnesium and zinc ions released into the PBS solution was determined by ICP-OES using an Optima 3100XL spectrometer (Perkin Elmer, Waltham, MA, USA).

## 3. Results

### 3.1. Physicochemical Properties of mHA Powder

**Figure 1:**
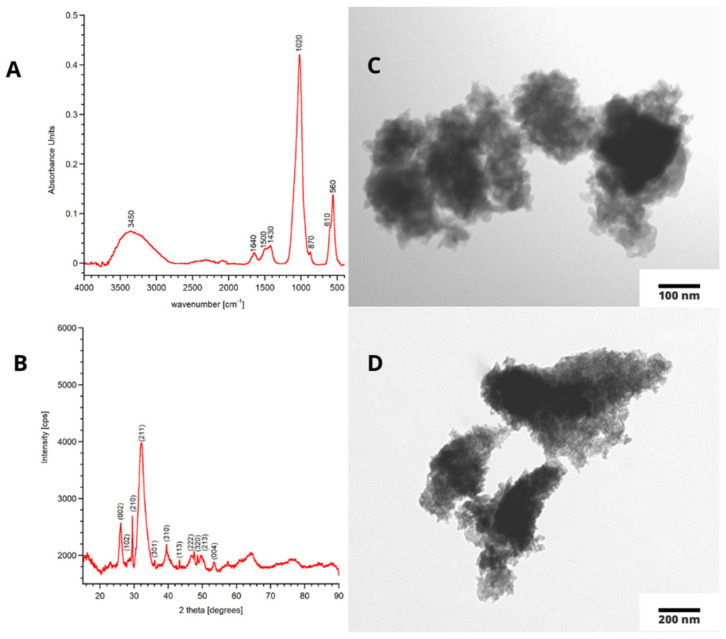
FT−IR spectrum of mHA-(**A**), PXRD diffractogram of mHA-(**B**), TEM images of mHA-(**C**,**D**).

The FT−IR spectrum of the obtained mHA powder is presented in Figure 1A. It exhibits the bands characteristic of orthophosphate ions at 1020 cm^−1^ and 560–610 cm^−1^, assignable to ν_1_ + ν_3_(PO_4_) and ν_4_(PO_4_), respectively. The wide bands at approximately 3450 cm^−1^ and 1640 cm^−1^ can be assigned to the stretching and bending vibrations, respectively, of the OH groups from physically adsorbed water. The bands in the 1550–1400 cm^−1^ region and a small band at 870 cm^−1^ refer to the CO_3_^2−^ groups. The curve-fitting process of the 1550–1400 cm^−1^ region (data not shown) depicts three main bands at 1510, 1450, and 1420 cm^−1^. The band at 1510 cm^−1^ belongs to carbonate type A (the substituting structural hydroxyl groups) and constitutes about 15% of the integral intensity of this region. The most intensive band at 1420 cm^−1^ (60% of the integral intensity) is typical in carbonates substituted for orthophosphates (type B), while the band at 1450 cm^−1^ refers to both types of carbonates. Thus, it can be concluded that carbonate ions present in the apatite structure are mainly located in place of phosphates.

The total content of carbonate ions calculated by the Clasen and Ruyter method was estimated to be 6.4 ± 0.3 wt.%, which is very close to the expected value (6.5%).

It is worth emphasizing that the large width of all bands present in the spectrum indicates the poorly crystalline nature of the material obtained. This is also evidenced by the weak separation of the phosphate bands, as well as the lack of a detectable band at 3570 cm^−1^, corresponding to the stretching vibrations of the structural OH groups.

The PXRD diffractogram of the mHA sample is shown in Figure 1B. The pattern reveals the reflections originating from only one crystalline phase of hydroxyapatite (ICDD 09-0432). 

As is clearly seen, the reflections are broad and poorly resolved, which is typical in poorly crystalline structures. The Scherrer formula was used to calculate crystallite sizes along the *a* and *c* axes, and the following result was obtained: the size of the crystallite along the *a* and *c* axes was 10.8 ± 0.5 and 6.5 ± 0.2 nm, respectively. It is worth mentioning that the crystal size of biological apatite had a great variety among reports (it is related to the age and location of bone tissue) from several nm to 100 nm [37].

The TEM images in Figure 1C,D indicate that the studied powder was nanosized with plate-like fine crystals. These results are in agreement with those from the FT-IR and PXRD methods described above. It is noteworthy that the needle-like or elongated crystals were not detected, which confirms the biomimetic morphology of the obtained powder [38]. A strong tendency for the crystals to form agglomerates can be observed; thus, it was impossible to use TEM images to measure crystal size.

In order to measure the zinc and magnesium content in the powder, the ICP-OES method was used. It is noteworthy that we aimed to synthesize a biomimetic HA that contained quantities of ions, similar to those found in biological apatite. Based on a literature review, the optimum value of Mg^2+^ was assumed to be approximately 0.5 wt.% [39]. It was also intended to introduce about 3.5% of the zinc ions to obtain additional osteogenic activity. The results provided from ICP-OES were as follows: 0.33 ± 0.02% and 3.21 ± 0.03% for magnesium and zinc ions, respectively. The zinc content is very similar to that assumed during synthesis, while magnesium content is slightly lower. Both ions (Zn^2+^ and Mg^2+^) feature smaller ion radii (0.074 and 0.072 nm, respectively) than Ca^2+^ (0.099 nm). According to the literature, magnesium substitution into hydroxyapatite is significantly limited (to approximately 0.5 wt.%) [40]. In turn, zinc ions may be introduced into hydroxyapatite crystals in two different ways, namely by either replacing calcium cations or by being inserted between two oxygen atoms in the columns of OH groups [41,42]. Nevertheless, it should be noted that these ions compete in the substitution of Ca^2+^ and, based on previous research, it can be assumed that zinc ions are more easily introduced into the HA crystal than Mg^2+^ ions [40,43].

The (Ca + Zn + Mg)/P molar ratio calculated using the ICP-OES results was 1.62 ± 0.02, which is typical for calcium-deficient hydroxyapatite [43].

### 3.2. Physicochemical Properties of Composites

To prepare collagen–apatite composites, apart from mHA powder, the following two types of collagen were used: atelocollagen and freeze-dried type I collagen from bovine Achilles tendon. 

**Figure 2:**
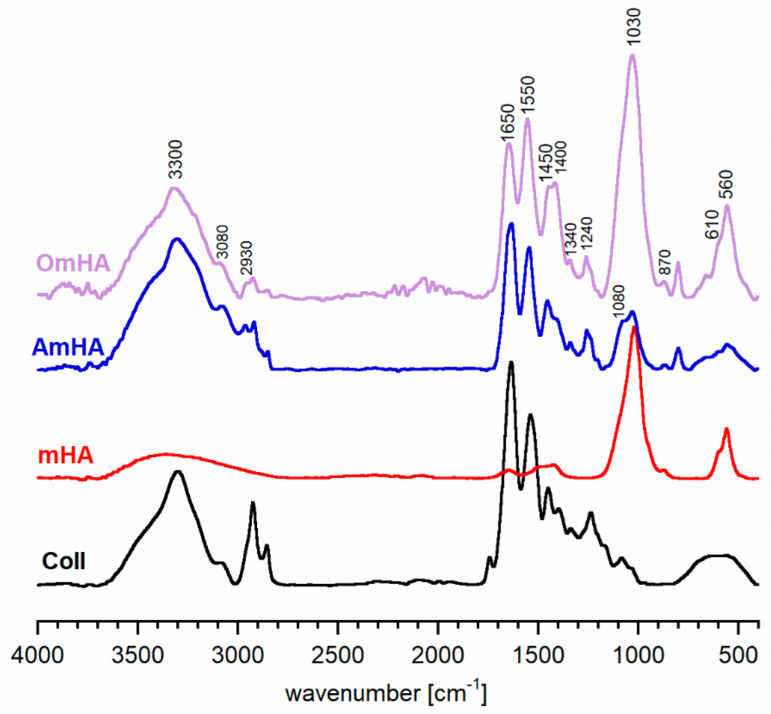
FT−IR spectra of the obtained composites (OmHA and AmHA), collagen type I (Coll), and hydroxyapatite (mHA).

FT-IR representative spectra of the obtained two types of composites, OmHA and AmHA, are shown in Figure 2. For comparison, mHA and collagen type I spectra are also presented in Figure 2. The spectra of OmHA and AmHA composites show the main characteristic bands of both mHA and collagen. The bands at 3300 cm^−1^ and 3080 cm^−1^ are typical for collagen protein and originate from N-H and C-H groups, respectively. The bands at 1650 cm^−1^ and at 1550 cm^−1^ can be assigned to C=O stretching for amide I and N-H deformation for amide II, respectively. The δ_CH_ bands are detectable in the 1450–1340 cm^−1^ region. The amide III band is observed at 1240 cm^−1^. It can be concluded that the preparation of composites did not influence the triple-helical structure of the collagen protein. 

In turn, the absorption bands at 1030 cm^−1^, 610 cm^−1^, and 560 cm^−1^ are attributed to the orthophosphate groups from the mHA fraction and are analyzed in detail in the Section 3.1.

Both OmHA and AmHA FT-IR spectra confirmed the inclusion of mHA in the collagen protein matrices. However, it can be clearly seen that the relative intensity of the mHA orthophosphate bands was significantly higher on the spectrum of the OmHA sample. It may be related to the more efficient adsorption of apatite nanocrystals on type I collagen than on atelocollagen fibers.

**Figure 3:**
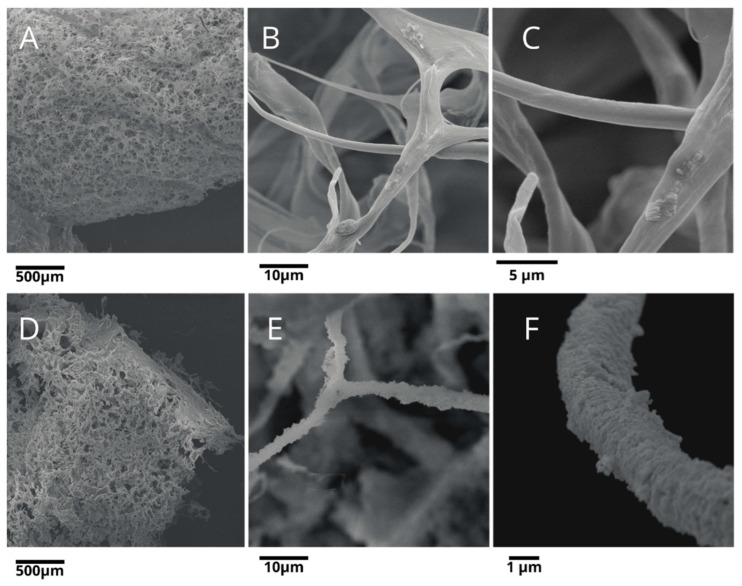
SEM images of the AmHA (**A**–**C**) and OmHA composites (**D**–**F**).

Figure 3 shows the SEM representative images of the OmHA and AmHA composites. Both samples exhibit a porous, sponge-like morphology (Figure 3A,D). There is a visible structure of irregular interconnected pores (Figure 3B,E). It can be observed that the pores are visible on the surface of the materials and in their internal structure. SEM results show that an interaction between the mHA nanocrystals and the collagen fibers occurred (Figure 3C,F). However, the scaffolds differed in the amount of mHA on the collagen matrix. Atelocollagen fibers were poorly embedded in mHA nanoparticles (AmHA), while collagen fibers (OmHA) were fully covered with small particles of mHA (see Figure 3C,F). SEM images indicate a better adsorption of mHA onto the collagen scaffolds with OmHA than with AmHA, which is in great accordance with the FT-IR results. 

### 3.3. Cytotoxicity Assay

BALB/c 3T3 cell viability did not decreased to under 70% in comparison to the untreated control by any of the tested materials. Therefore, all samples were classified as not cytotoxic in the neutral red uptake assay (see Table 2). Moreover, none of the materials negatively affected the morphology of the culture. Fibroblasts after exposition of the tested samples did not differ from the control cultures incubated in a fresh medium.

### 3.4. Drug and Ions Release

**Figure 4:**
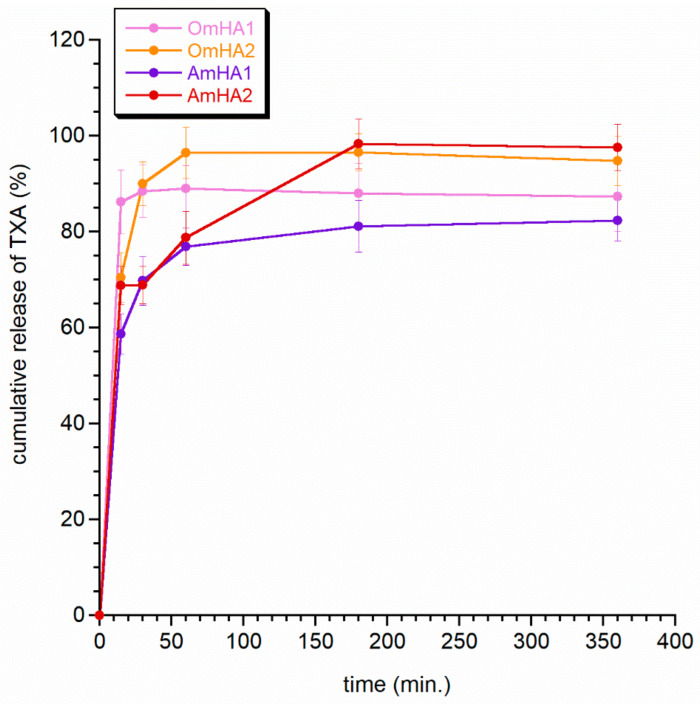
TXA release profiles from the obtained composites.

An anti-hemorrhagic drug, TXA, was introduced into the composite OmHA and AmHA structure by the following two different methods: directly into mHA and protein suspension (yielding the samples OmHA1 and AmHA1) before lyophilization, and by soaking the lyophilized composites in the TXA solution (yielding the samples OmHA2 and AmHA2). In Figure 4, the cumulative release of TXA curves for the four composites is presented. As shown, TXA was rapidly released from all obtained composited during the first hour and took the form of burst release profiles. More than 58% of the initially loaded drug was released in 15 min. The OmHA1 composite release profile exhibits the highest burst release effect: after 15 min, the release rate reached 86%. The AmHA1 and OmHA2 composites had similar drug release profiles: the maximum amount of cumulative release over the measured time occurred after 180 min and reached 97 and 81% for OmHA2 and AmHA1, respectively.

The sample AmHA2 is characterized by a slightly different release profile (see Figure 4). In the first stage of TXA release, the effect of burst release can be easily distinguished, followed by a slightly slower release of TXA after 30 min. The curve reached the plateau after 180 min, similar to the OmHA2 and AmHA1 curves.

**Figure 5:**
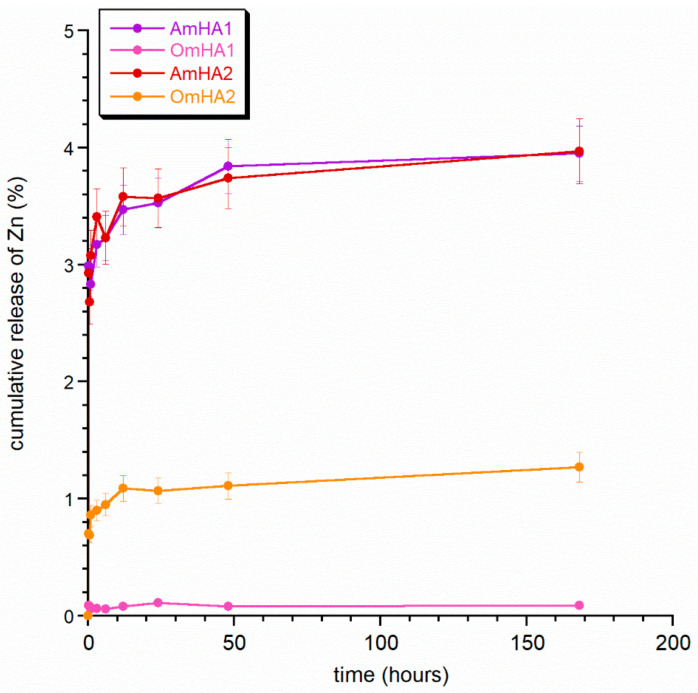
Release of zinc ions from the obtained composites.

**Figure 6:**
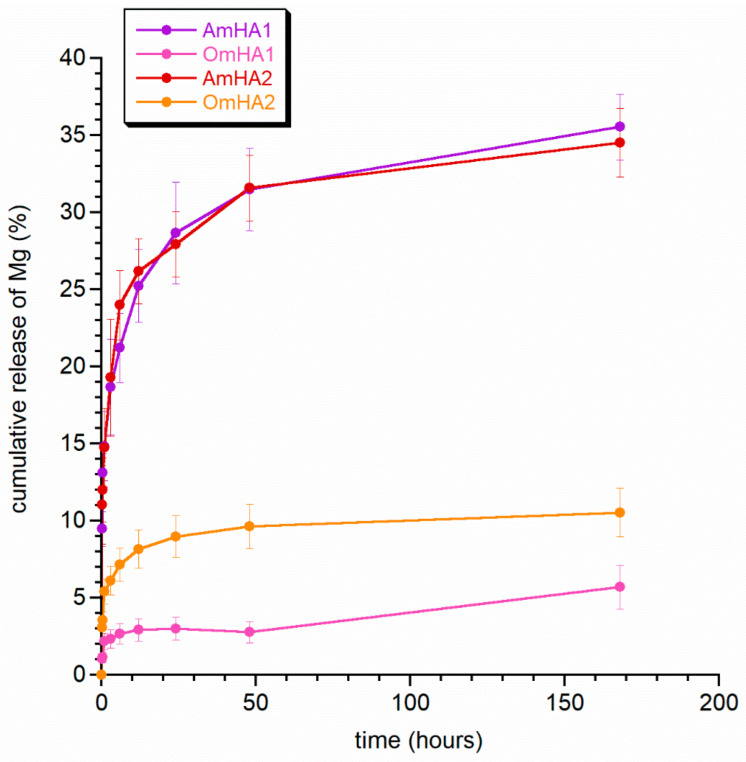
Release of magnesium ions from the obtained composites.

The study shows that TXA released rapidly, intending to achieve a hemostatic effect as quickly as possible. Overall, the observed differences in the release period were not significant and were probably the result of both the preparation of the composites and the incorporation of the drug into them. This requires further in-depth research. The next step was to observe the release profile of zinc and magnesium from the mineral fraction of composites (see Figure 5 and Figure 6). Both figures clearly show that the ions released easier from the AmHA1 and AmHA2 than from the OmHA1 and OmHA2 samples. This is most likely related to the structure of these composites and the significantly weaker bonds of mHA with atelocollagen fibers. mHA, being loosely packed in the AmHA fiber matrix, easily “escapes” from it, which facilitates the release of zinc and magnesium ions, especially those located on the surface of agglomerated mHA nanocrystals.

Interestingly, in the studied period, the release of zinc ions was negligible from each of the samples (in samples OmHA1 and OmHA2, it did not exceed 1.5%, while in samples AmHA1 and AmHA, it did not exceed 2.4%). 

It can therefore be assumed that Zn^2+^ ions are mainly located in the crystalline structure of apatite and that they are not released during this time as a result of apatite resorption. On the other hand, the release of Mg^2+^ is significantly higher. For samples AmHA1 and AmHA2, approximately 35% of magnesium was released within 7 days, while for samples OmHA1 and OmHA2, this value did not exceed 11%. 

There is an observed higher degree of release of magnesium ions in all the composites compared to the % of released zinc. According to the literature, the incorporation of magnesium ions into the structure of hydroxyapatite by calcium substitution is much more difficult than that of zinc ions. What is more, the substitution limit of magnesium ions is quite low, amounting to approx. 0.5%, while zinc ions are incorporated in significant amounts and, at the same time reduce the crystallinity of apatite. It can therefore be assumed that zinc hinders the substitution of calcium by magnesium ions, which, consequently, are partially deposited on the surface of nanocrystals in the so-called hydrated surface layer. Thus, they are more easily released from mHA and its composites.

The ion release curves did not plateau, which is of course in line with the literature data on long apatite resorption over a period of at least a few weeks.

**Figure 7:**
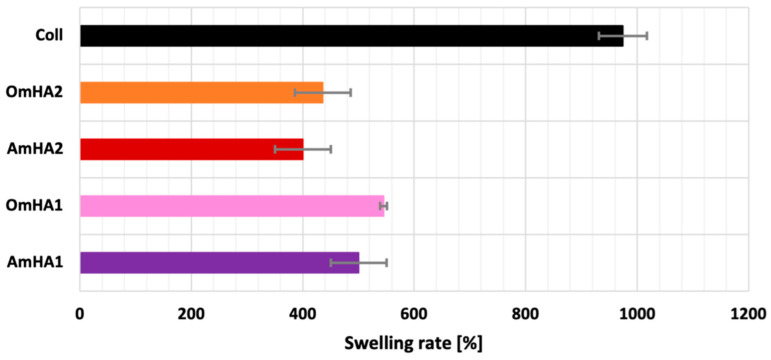
Results of swelling test for the obtained composites and collagen type I.

Figure 7 presents swelling studies of the obtained composites. The results indicate a very high ability in water absorption of all the samples. The water uptake ratio rapidly increased within the first 30–60 min and reached 400 to 1000%. 

The addition of mineral phase (mHA) in the collagen (both atelocollagen and collagen type I) decreased the swelling degree of samples. This could be a result of the replacement of collagen with apatite that did not absorb as much water as collagen matrices [44]. It can be assumed that the addition of mHA to the composites led to the limitation of swelling, most likely due to collagen–mHA interactions [44,45]. However, it is worth emphasizing that, despite this, water absorption was still at a high level.

## 4. Conclusions

Herein, we described the fabrication of porous composites for their potential use in dental surgery. We successfully fabricated modified hydroxyapatite/collagen composites containing tranexamic acid as an antihemorrhagic drug. All formulated composites revealed substantial physicochemical and morphological features. The OmHA composites exhibited significant homogeneity, and the mHA nanocrystals almost completely covered collagenous fibers. Preliminary in vitro cytotoxicity tests classified all samples as not cytotoxic. Our studies showed that TXA was released very quickly from each type of composite, while Mg and Zn ions were characterized by a significantly slower release. It should be noted that the 3D porous composite design still requires improvement.

In our opinion, the research presented in this paper is preliminary but promising enough to be continued in an in vivo model.

In vivo studies would provide an opportunity to check whether tranexamic acid released rapidly from the composite matrices would have a beneficial effect, despite the inhibition of fibrinolysis. Of course, it will also be important to study wound healing and bone growth in both a basic animal model and in various conditions that affect wound healing, such as type 2 diabetes.

It would also be very interesting to check the effect of the release of zinc and magnesium ions on the process of new bone formation.

Therefore, mechanical properties tests, the optimization of ion release, and in vivo studies will be the focus of our future research.

## Figures and Tables

**Table 1:** Types of composites.

Sample	Collagen Type	TXA Addition Method
**AmHA**	atelocollagen from bovine dermis	-
**OmHA**	collagen type I from bovine Achilles tendon	-
**AmHA1**	atelocollagen from bovine dermis	direct addition of TXA powder before freeze-drying
**AmHA2**	atelocollagen from bovine dermis	soaking in a TXA solution
**OmHA1**	collagen type I from bovine Achilles tendon	direct addition of TXA powder before freeze-drying
**OmHA2**	collagen type I from bovine Achilles tendon	soaking in a TXA solution

**Table 2:** Results of the neutral red uptake test for the highest concentrations of tested extracts [50 mg/mL] in comparison to the untreated control. The test was conducted for up to 24 h.

Sample	Cell Viability ± SD [%]
mHA	102 ± 1
AmHA	107 ± 5
OmHA	97 ± 5
LT	0 ± 0
PE	102 ± 1

LT—latex, reference cytotoxic material. PE—polyethylene foil, reference non-cytotoxic material.

## Data Availability

Not applicable.

## Abbreviations

Coll	collagen type I
DMEM	Dulbecco’s modified eagle medium
FT-IR	Fourier-transform infrared spectroscopy
HA	hydroxyapatite
HPLC	high-performance liquid chromatography
ICP-OES	inductively coupled plasma–optical emission spectroscopy
mHA	mimetic hydroxyapatite
PBS	phosphate-buffered saline
PTX	parathyroid hormone
PXRD	powder X-Ray diffraction
SEM	scanning electron microscopy
TEM	transmission electron microscopy
TXA	tranexamic acid
